# Organized Sports and Physical Activities as Sole Influencers of Fitness: The Homeschool Population

**DOI:** 10.3390/jfmk4010013

**Published:** 2019-01-28

**Authors:** Laura S. Kabiri, Augusto X. Rodriguez, Amanda M. Perkins-Ball, Cassandra S. Diep

**Affiliations:** Kinesiology Department, Rice University, Houston, TX 77005, USA

**Keywords:** sport, conditioning, physical activity, children, adolescents

## Abstract

Homeschool children may rely solely on organized sports and physical activities to achieve recommended levels of physical activity and fitness. The purpose of this study was to investigate differences in fitness levels between homeschool children who did, and did not, participate in organized sports or physical activities, and then examine relationships between hours per week in sports or physical activities and cardiorespiratory fitness as measured by portions of the FitnessGram^®^ test battery. Organized sports/physical activity participation information was gathered on 100 children ages 10–17 years who completed tests of upper, abdominal, and cardiorespiratory fitness. The current investigation revealed that participation alone was not associated with higher levels of physical fitness as assessed by the 90° push-up test or curl-up test nor was time in participation related to cardiorespiratory fitness as assessed by the Progressive Aerobic Capacity Endurance Run (PACER). These activities alone may be insufficient for meeting physical activity recommendations and improving physical fitness. Therefore, children and adolescents educated at home may need additional opportunities to participate in unstructured daily physical activity.

## 1. Introduction

Physical activity and fitness are critical for health and wellbeing. Specifically, participation in organized sports is a way for children and adolescents to get the recommended levels of physical activity [1] and has been recommended by the American Academy of Pediatrics as an opportunity for children and adolescents to increase their physical activity [2]. Defined as “an activity involving physical exertion and skill in which an individual or team competes against another or others for entertainment” [3], sports may include moderate (e.g., badminton, cricket) or vigorous-intensity physical activity (e.g., competitive swimming) [4]. Numerous studies have found sports participation to be beneficial for children and adolescent’s psychological, social, and physical health [5,6,7,8,9,10,11,12].

A systematic review of the psychological and social benefits of participation in sports among youth revealed higher self-esteem, fewer depressive symptoms, higher confidence, and improved teamwork and social skills among sport participants than non-sport participants [5]. Sports participation was also related to beneficial health behaviors (e.g., fruit and vegetable consumption) [6], more positive attitudes and beliefs about physical activity [7], and physical activity levels [6]. These benefits may carry into adulthood, as sports participation in childhood and adolescence has been associated with physical activity levels in adulthood [13,14,15,16].

Cardiorespiratory and muscular fitness may also be improved with sports participation. In one study, U.S. adolescents who played school sports performed more pull-ups, had stronger grip strength, and performed the plank fitness test longer than those not in school sports [5]. School sports participation was also associated with improved performance on the 20-m shuttle run in a sample of Australian youth [8]. Outside of school, participation in sports outside school and participation in sports competitions were associated with better performances on the 20-m shuttle run in a sample of youth in Portugal [9,10]. Children from Denmark, who participated in gymnastics, handball, tennis, and swimming, had high levels of anaerobic power and muscular strength [11]. Another study of ninth-grade girls in the U.S. specifically found that, those who participated on at least two sports teams (either in school or outside school), performed better on the step test than those who did not participate in any sports [12]. Further, adolescent females in cycling, running, and swimming [17] and adolescent males in various high-impact sports (e.g., football, rugby, and hockey) [18] had higher bone mineral density.

Despite existing research on the benefits of participation in organized sports among children and adolescents, one population that has been overlooked in such research is homeschool children and adolescents, who may be at increased risk for cardiovascular disease and adiposity [19]. Unlike public school children, homeschool children do not regularly participate in school-based physical activity (e.g., physical education, recess, school sports) because they are not subject to state regulations for physical education classes, physical activity initiatives, or fitness testing [19]. Thus, organized sports and organized physical activities may be the only avenues through which homeschool children engage in purposeful exercise.

With an increasing prevalence of homeschooling and home education around the world (including almost 2 million children in the U.S. [19]), and the lack of regulations for physical education, research is needed to investigate whether organized sports and physical activities alone are sufficient to improve fitness in homeschool youth. Thus, the purpose of this study was to investigate differences in fitness levels between homeschool children who did and did not participate in organized sports or physical activities. A secondary purpose was to examine any relationships between average hours per week in sports or physical activities and cardiorespiratory fitness. Given previous research on the relationship between sports and fitness, we hypothesized that homeschool children who participated in organized sports or physical activities would have improved fitness over those who did not participate in organized sports or physical activities, and that more hours would be related to improved cardiorespiratory fitness. 

## 2. Materials and Methods

Participant recruitment and data collection occurred after ethical approval from the Institutional Review Board (Protocol #18919 and #19736) of Texas Woman’s University on 3 March 2016 and 19 January 2017 respectively as part of Fitness Assessment in the Homeschool: The F.A.I.T.H. Study—Part I and II. Homeschool children ages 5–17 years were recruited from the Greater Houston area through email, homeschool groups, and word of mouth. A subset of this population (ages 10–17 years) was intended for use in this study due to lack of normative data for younger children on selected outcome measures. An *a priori* power analysis with an alpha of 0.05, power of 0.8, and effect size of 0.3 revealed a necessary sample size of 88 participants.

Parents completed a survey including information on whether their child was currently participating in organized sports or physical activities (yes/no). If the answer was yes, the parent was asked to provide the average number of hours of participation per week. For the purposes of this study, organized participation was defined as any sport or physical activity in which the child paid to participate. 

To assess multiple aspects of physical fitness, all participants completed the 90° push-up test, curl-up test, and Progressive Aerobic Capacity Endurance Run (PACER) as part of the FitnessGram^®^ test battery (v. 10.0; Human Kinetics, Champaign, IL, USA). The 90° push-up test is a measure of upper body strength and endurance while the curl-up test assesses abdominal strength and endurance. The PACER is a test of cardiorespiratory fitness and is administered similar to the 20-m shuttle run or 20-m beep test [20]. The FitnessGram^®^ test battery has been shown to be both reliable and valid in this population and is routinely employed in American public schools [21,22,23]. All tests were administered as per the FitnessGram^®^ administration manual and performed until two failed repetitions or volitional exhaustion, whichever occurred first [20].

Results for each test portion (90° push-up, curl-up, PACER) were dichotomized into healthy or needs improvement classifications. The PACER was also used to calculate an age and gender specific estimated VO_2max_ for each participant to measure cardiorespiratory fitness in addition to the dichotomized classification. All results and classifications were calculated using age and gender specific normative data provided by FitnessGram^®^. This was done to account for the effects of both age and gender on test results.

Chi-square tests were used to explore statistically significant differences in fitness between children who did and did not participate in organized sports or physical activities. Comparisons were made between participation groups for overall fitness (healthy rating for all three tests) as well as for a healthy classification on each individual test (90° push-up, curl-up, PACER). Pearson correlation coefficient was used to determine any relationship between average hours per week of organized sports or physical activity and cardiorespiratory fitness (VO_2max_). All statistical analyses were done using IBM SPSS Statistics for Windows (v. 25.0; IBM, Corporation, Armonk, NY, USA) with an alpha level of *p* = 0.05 used to indicate statistical significance. 

## 3. Results

### 3.1. Participant Demographics

A total of 211 participants aged 5–17 years enrolled in the study. Of those, 100 participants met the age requirement (10–17 years) for this portion of the study. This subset (*n* = 100) of age-appropriate participants was used for all data analyses. Participant characteristics can be found in Table 1; Table 2. The sample was evenly split between genders with an average age of 12.71 years. They were predominantly non-Hispanic white and of normal body mass index.

### 3.2. Outcomes

Overall, 80% of participants (*n* = 80) were currently engaged in some form of organized sports participation or physical activity. Healthy classification overall and for each individual test can be seen in Table 3 while specific test performance details are in Table 2. The sample had a mean of 4.68 h/week of sports participation (Range = 0–17; SD = 3.60) with a majority exhibiting good upper body strength and endurance as well as cardiorespiratory fitness. 

Chi-square tests revealed no significant (χ^2^ (1, *n* = 100) = 0.903 *p* = 0.342.) difference between participation groups for overall fitness as seen in Table 4. These results indicate no association between participating in organized sports or physical activity and being in the healthy fitness zone in all three categories. Additional chi-square tests found similar non-significant differences between groups for a healthy classification on each individual test as well (90° push-up: χ^2^ (1, *n* = 100) = 0.490, *p* = 0.484; curl-up: χ^2^ (1, *n* = 100) = 0.526, *p* = 0.468; PACER: χ^2^ (1, *n* = 100) = 0.005, *p* = 0.942). Pearson correlation revealed a non-significant relationship between average hours per week of organized sports or physical activity and estimated VO_2max_ (*r* = 0.121, *p* = 0.230).

## 4. Discussion

The primary findings of this study demonstrated no relationship between participation in organized sports and physical fitness among homeschool children and adolescents. The majority of subjects participated in some form of organized sport or physical activity. However, participants did not have higher levels of overall fitness (i.e., achieved a healthy rating for all three tests) or for each individual test (i.e., 90° push-up, curl-up, PACER) than non-participants. Furthermore, there was no relationship between number of hours spent participating in sport each week and cardiorespiratory fitness. These findings were in direct opposition to our original hypotheses.

Previous studies have provided evidence supporting the relationship between sport participation and physical fitness indicators among children and adolescents [8]. High levels of cardiorespiratory endurance, muscular strength and endurance, and power among girls and boys participating in a variety of sports, including tennis, gymnastics, handball, and swimming, have been well documented [10,11]. Further, studies examining running and high-volume loading sports, such as soccer, basketball, tennis, and rugby, have been associated with higher bone mineral density [17,18].

While previous research has found positive associations between sport and fitness, the results of our study suggest sport participation alone may not be enough to achieve desired levels health-related physical fitness. This may be because the achievement of physical activity recommendations is important for developing physical fitness [24]. The World Health Organization (WHO) suggests children and youth ages 5–17 accumulate a minimum of 60 min of primarily aerobic moderate—to vigorous-intensity physical activity (MVPA) daily, as well as muscle- and bone-strengthening activities at least three days per week, in order to improve health and physical fitness [25].

Participation in organized sport alone may be insufficient for children in order for them to meet daily physical activity guidelines for several reasons, including not participating in MVPA outside of sport practices, not practicing frequently, or practices being low in MVPA [26]. Recent analyses of youth sport practices revealed that children engaged in MVPA 34–50% of their time spent in practice [26,27,28,29], which is approximately 20–30 min (one-third to one-half) of MVPA toward daily public health guidelines [26,27]. When exploring varied practice structures and time segments in youth flag football, Schleter and colleagues [27] found that free-play, game-play, and warmup segments resulted in greater percentages of time spent in MVPA than scrimmage, strategy, and sport-skill segments of practice. A number of contextual variables may contribute to low amounts of MVPA during a practice session, such as those related to tasks (e.g., time devoted to organizational tasks, strategy, or self-care), and setting (e.g., fewer opportunities to participate in relation to children available to participate) [27]. Recent interventions have implemented strategies in effort to address these factors to increase MVPA during youth sport practices [29,30].

To the best of our knowledge, no previous studies have examined the relationship between sports participation and achievement of health-related physical fitness among homeschool children and adolescents. Strengths of the study include a sample size exceeding the required number of participants determined by an a priori power analysis, as well as the broad age range and even gender representation of our population. Recruitment of children not currently participating in physical education classes through the public-school system also strengthens our findings by focusing solely on effects of organized sports and activities. Limitations of the study include assessment of organized sports and physical activity participation by parental report only and failure to further qualify or classify the type of participation. In addition, participants consisted of majority healthy weight children and adolescents; therefore, the sample did not allow the researchers to control for obesity, a known confounding variable. Further, the study did not quantify participants’ habitual physical activity. Previous studies investigating the effects of habitual physical activity on fitness among children and adolescents have yielded mixed results [31]. Future studies should attempt to more accurately quantify the amount of sport practice time spent engaged in MVPA.

## 5. Conclusions

The current investigation revealed that organized sport participation and/or physical activity alone was not associated with higher levels of physical fitness among 10–17 year old homeschool students. These activities alone may be insufficient for meeting MVPA recommendations and improving physical fitness. Therefore, children and adolescents educated at home may need additional opportunities to participate in unstructured physical activity daily.

## Figures and Tables

**Table 1:** Frequencies for participant characteristics.

Variable	*n*	Percent
**Gender**		
Male	50	50%
Female	50	50%
Total	100	100%
**Ethnicity**		
Non-Hispanic White	83	83%
Hispanic	5	5%
Non-Hispanic Black	5	5%
Asian	3	3%
Other	4	4%
Total	100	100%

Note: *n*: 100.

**Table 2:** Descriptive characteristics for physiological characteristics.

**Variable**	***n***	**Mean**	**SD**
Age (years)	100	12.71	2.17
Years in homeschool	100	5.80	2.85
Hours per week in sports participation	100	4.68	3.60
Curl-up repetitions	100	15.82	15.80
Push-up repetitions	100	16.47	8.078
PACER laps	100	35.16	18.08
Estimated VO_2max_	100	43.78	6.23
**BMI Classification**	***n***	**Percent**	
Under weight	3	3%	
Normal weight	81	81%	
Overweight	10	10%	
Obese	6	6%	
Total	100	100%	

Note: *n*: 100.

**Table 3:** Sports participation and healthy status frequencies.

Sports Participation	*n*	Percent
Yes	80	80%
No	20	20%
Total	100	100%
**Overall Healthy Classification**		
Unhealthy in at least one	77	77%
Healthy in all three	23	23%
Total	100	100%
**Curl-up Healthy Classification**		
Needs improvement	63	63%
Healthy	37	37%
Total	100	100%
**Push-up Healthy Classification**		
Needs improvement	15	15%
Healthy	85	85%
Total	100	100%
**PACER Healthy Classification**		
Needs improvement	33	33%
Healthy	67	67%
Total	100	100%

Note: *n*: 100.

**Table 4:** Crosstab analysis.

**Variable**	**No**	**Yes**	**Total**
Unhealthy in one	17	60	77
Healthy in all	3	20	23
Total	20	80	100
	**Value**	**df**	**Sig.**
Pearson Chi-Square	0.903	1	0.342

Notes: df (degree of freedom): 1; Sig.: *p* = 0.05.
